# Mapping the Translational Research Structure of Photobiomodulation in Osteoarthritis: A Bibliometric Analysis

**DOI:** 10.3390/bioengineering13070811

**Published:** 2026-07-15

**Authors:** Minh Le Tran, Ji-Woo Seok

**Affiliations:** 1 Digital Health Research Division, Korea Institute of Oriental Medicine, Daejeon 34054, Republic of Korea; minhletran@kiom.re.kr; 2 Korean Convergence Medical Science, University of Science and Technology, Daejeon 34113, Republic of Korea

**Keywords:** photobiomodulation, osteoarthritis, bibliometric analysis, translational research, regenerative medicine, rehabilitation

## Abstract

**Background:** Osteoarthritis (OA) is a complex whole-joint disease imparting a substantial global socioeconomic burden. Photobiomodulation (PBM) has attracted increasing research interest as a non-pharmacological intervention for OA, but the intellectual structure and developmental trajectory of this research field remain incompletely understood. **Objective:** This study systematically mapped the intellectual landscape, research hotspots, thematic structure, and translational characteristics of PBM research in OA using bibliometric methods. **Methods:** Bibliographic records were retrieved from the Web of Science Core Collection database. Following data cleaning, 422 publications (1988–2026) were analyzed using the bibliometrix package in R and VOSviewer software. **Results:** Publication output increased substantially after 2015, with a marked rise after 2020. Keyword co-occurrence analysis classified 71 core keywords into seven clusters, revealing a dual-axis knowledge structure comprising a mechanistic biology axis (inflammation, chondrocytes, oxidative stress, and cartilage) and a clinical rehabilitation axis (pain, WOMAC, exercise, and physical therapy). Overlay visualization and thematic map analyses indicated a gradual shift in research focus from symptom-oriented rehabilitation research toward mechanistic investigations and regenerative medicine-related approaches involving platelet-rich plasma and mesenchymal stem cells. Reference co-citation analysis identified two major citation clusters connected through studies related to inflammation, pain management, and rehabilitation. **Conclusions:** The PBM-OA literature is characterized by a translational knowledge structure integrating mechanistic biology and rehabilitation-oriented research. Notably, recent publication trends indicate increasing scholarly attention to regenerative medicine-related approaches while continuing to position PBM within exercise-centered conservative management. To strengthen the evidence base and guide future investigations, future research should prioritize protocol standardization, dose–response validation, and long-term structural outcomes.

## 1. Introduction

Osteoarthritis (OA) represents one of the most prevalent chronic musculoskeletal disorders globally, serving as a primary driver of chronic pain, joint stiffness, functional limitations, and a subsequent decline in quality of life [1,2,3]. While historically categorized as a simple degenerative joint disease, OA is now increasingly recognized as a complex whole-joint disorder. This pathology encompasses the entire articular organ, characterized not only by progressive cartilage degradation but also by subchondral bone remodeling, synovitis, osteophyte formation, and periarticular alterations [2,3,4]. These multifaceted pathophysiological changes inevitably induce chronic pain and severe functional disability, progressively compounding the global socioeconomic burden of OA in an aging society [1,2,5].

Currently, clinical guidelines recommend non-surgical management, centered on patient education, self-management, weight control, and physical exercise, as the core foundational therapy for OA, while pharmacological interventions and intra-articular injections are utilized as adjunctive modalities for symptom palliation [1,2]. However, definitive disease-modifying osteoarthritis drugs (DMOADs) capable of fundamentally halting disease progression or restoring damaged joint tissues remain profoundly limited [2,3]. Consequently, there is a burgeoning interest in novel, non-pharmacological therapeutic strategies that safely and effectively target the underlying pathophysiology of OA [3,4].

Against this backdrop, photobiomodulation (PBM) has attracted increasing research interest as a non-invasive intervention for OA [3,4,6]. PBM involves the application of red or near-infrared (NIR) light to biological tissues to induce photochemical reactions, modulating biological responses at the cellular level without generating thermal ablation [4,6]. Mechanistically, mitochondrial cytochrome c oxidase acts as the primary photoacceptor, triggering a cascade of intracellular events including enhanced adenosine triphosphate (ATP) synthesis, nitric oxide (NO) release, redox regulation, and the activation of downstream signaling pathways [2,4]. These biological responses are closely associated with several key pathophysiological mechanisms underlying OA, such as chronic inflammation, oxidative stress, chondrocyte dysfunction, and extracellular matrix (ECM) degradation. Recent experimental and clinical studies have increasingly investigated PBM in relation to chondroprotection, tissue repair, and regenerative medicine-related mechanisms [2,3,6].

Indeed, the volume of literature investigating PBM in the context of OA has increased substantially over the past two decades [3,5]. While initial investigations predominantly focused on validating the clinical efficacy of PBM regarding pain reduction and functional recovery [7], the subsequent accumulation of randomized controlled trials (RCTs), systematic reviews, and meta-analyses has contributed to an expanding body of clinical and preclinical literature [3,7]. Concurrently, preclinical studies have elucidated various microscopic mechanisms, including the modulation of pro-inflammatory cytokines, attenuation of oxidative stress, protection of chondrocytes, preservation of the ECM, and stimulation of tissue regeneration [4,6]. More recently, the scope of research has broadened further to explore convergence with regenerative medicine, incorporating platelet-rich plasma (PRP), mesenchymal stem cells (MSCs), advanced biomaterials, and innovative intra-articular photobiomodulation paradigms [2].

Despite this rapid proliferation of literature, existing studies remain largely fragmented, focusing heavily on isolated clinical efficacy trials, specific treatment protocols, or localized biological mechanisms [3,4,6]. Consequently, a comprehensive macro-level analysis encompassing the overall intellectual structure, shifting research hotspots, evolutionary trajectories, and the translational relationship between mechanistic benchmarks and clinical applications within the PBM-OA field remains visibly scarce. Furthermore, the manner in which emerging regenerative medicine paradigms are connected to and evolving from traditional rehabilitation-oriented research has yet to be systematically characterized [2].

To address these knowledge gaps, the present study sought to systematically map the intellectual landscape and evolutionary dynamics of PBM research for OA utilizing advanced bibliometric and scientometric methodologies. Specifically, we analyzed publication trends, keyword co-occurrence networks, chronological research flows, thematic structures, and reference co-citation architectures to identify core research themes and foundational knowledge bases. Ultimately, this study aims to characterize the intellectual structure, thematic evolution, and research landscape of PBM research in osteoarthritis, with particular emphasis on how mechanistic, rehabilitation, and regenerative medicine-related themes have developed within the published literature.

## 2. Methods

### 2.1. Data Source and Search Strategy

This study conducted a bibliometric analysis based on the Web of Science Core Collection (WoSCC) database to characterize the intellectual structure and research trends in the field of photobiomodulation (PBM) and osteoarthritis. The literature search was performed on 24 May 2026.

The search was conducted in the Topic field (TS), which searches the title, abstract, author keywords, and Keywords Plus, using the following query: TS = ((“photobiomodulation” OR “photobiomodulation therapy” OR “low level laser therapy” OR “low-level laser therapy” OR LLLT OR PBM OR PBMT OR “laser therapy” OR “low power laser” OR phototherapy) AND (osteoarthritis OR “knee osteoarthritis”)). No restrictions were applied with respect to publication year or language. Document types were subsequently restricted to articles and review articles.

### 2.2. Data Cleaning and Preprocessing

The retrieved bibliographic records were converted into a bibliographic dataframe using the bibliometrix package in R. Subsequently, duplicate records were identified based on document titles, and no duplicate publications were detected.

During data preprocessing, PBM- and osteoarthritis-related terms were identified from titles, abstracts, author keywords (DE), and Keywords Plus (ID). All keywords were converted to lowercase and standardized for punctuation, spacing, and abbreviations prior to keyword normalization. To improve data consistency, keyword normalization, synonym merging, and noise filtering were performed. Specifically, various PBM-related expressions, including “low-level laser therapy”, “LLLT”, “PBMT”, and “laser therapy”, were standardized as “photobiomodulation”. Similarly, osteoarthritis-related terms such as “knee osteoarthritis”, “osteoarthrosis”, and “degenerative joint disease” were normalized as “osteoarthritis”. Unrelated disease terms (e.g., rheumatoid arthritis) were excluded. In addition, generic intervention terms (e.g., “therapy”, “treatment”, and “management”), overly broad descriptors (e.g., “disease” and “review”), and technical or non-conceptual terms that did not represent distinct research topics were removed to improve the interpretability of the keyword co-occurrence network. Duplicate keywords within individual records were also removed after normalization. The complete keyword normalization and filtering scheme is provided in Supplementary Table S1.

In addition, a VOSviewer thesaurus file and user-defined cleaning functions were applied to integrate synonyms and remove irrelevant keywords. The resulting cleaned dataset (PBM_OA_clean_dataset) was used for all subsequent bibliometric analyses.

### 2.3. Bibliometric Analysis

Bibliometric analyses were conducted using the bibliometrix package (v5.4.1) in R software (version 4.5.2). Bibliometric indicators, including annual publication trends, leading journals, author keyword frequencies, and Keywords Plus frequencies, were calculated. An overall bibliometric summary of the dataset was generated using the biblioAnalysis() and summary() functions.

### 2.4. Keyword Co-Occurrence Network and Temporal Analysis

Keyword co-occurrence network analysis was performed using the biblioNetwork() and networkPlot() functions of the bibliometrix package. Both author keywords (DE) and Keywords Plus (ID) were included in the analysis. Association strength normalization and the Fruchterman–Reingold layout algorithm were applied to construct the network. This enabled the identification of major research clusters and relationships among core keywords.

Network, overlay, and density visualizations were further generated using VOSviewer software (version 1.6.20; Leiden University, Leiden, The Netherlands). A thesaurus file was applied to merge synonymous terms and remove noise keywords, while the full counting method and association strength normalization were employed (Table S2). Synonymous keywords referring to the same concept (e.g., “low-level laser therapy (LLLT)”) were merged into the standardized term “photobiomodulation” in accordance with the international consensus on PBM nomenclature to prevent artificial fragmentation of conceptually identical terms. Generic, non-informative, and non-target keywords were removed because they did not represent distinct research themes. These preprocessing procedures improved the interpretability of keyword co-occurrence networks and thematic maps by ensuring that each node represented a unique research concept rather than variations in terminology. To optimize network readability and structural stability, several candidate minimum occurrence thresholds were iteratively evaluated, and a minimum occurrence threshold of five was ultimately selected, resulting in a final network comprising 71 keywords.

Overlay visualization was used to assess the temporal evolution of research topics based on the average publication year associated with each keyword. Density visualization was used to identify major research hotspots characterized by high occurrence frequencies and strong network connectivity.

### 2.5. Thematic Map Analysis

The thematic structure and developmental maturity of the PBM-OA research field were evaluated using the thematicMap() function of the bibliometrix package. The analysis was based on Keywords Plus (ID), and themes were classified into motor themes, basic themes, niche themes, and emerging or declining themes according to their centrality and density. This classification enabled an assessment of the relative importance and developmental status of individual research themes.

### 2.6. Reference Co-Citation Network Analysis

Reference co-citation network analysis was conducted to investigate the intellectual structure of the PBM-OA research field. Co-citation networks were constructed using the biblioNetwork() function of the bibliometrix package based on co-citation relationships among cited references.

The resulting network was visualized using the networkPlot() function to identify major co-citation clusters and the intellectual backbone of the field. This analysis facilitated the exploration of intellectual connections between preclinical mechanistic studies and clinical rehabilitation research within the PBM-OA literature.

## 3. Results

### 3.1. Overview of Included Publications

The initial search in the Web of Science Core Collection (WoSCC) database yielded a total of 503 publications. After restricting the document types to original articles and review articles, 433 publications were selected, with no duplicate records identified based on document titles (*n* = 0). Following data cleaning and preprocessing, a final set of 422 publications was included in the analysis (Figure S1). The publication years of the analyzed literature ranged from 1988 to 2026. Regarding document types, original articles accounted for 305 papers (72.3%), while review articles accounted for 117 papers (27.7%) (Table S3). The dataset comprised 198 publication sources, 2212 authors, and 14,873 cited references. The average number of authors per publication was 6.24, and international co-authorship was observed in 24.64% of publications. Furthermore, 366 publications (86.7%) were classified as preclinical/mechanistic research, whereas 56 publications (13.3%) were classified as clinical/rehabilitation research, indicating a marked predominance of preclinical and mechanistic studies (Figure S2).

### 3.2. Publication Trends and Leading Journals in PBM–Osteoarthritis Research

**Figure 1:**
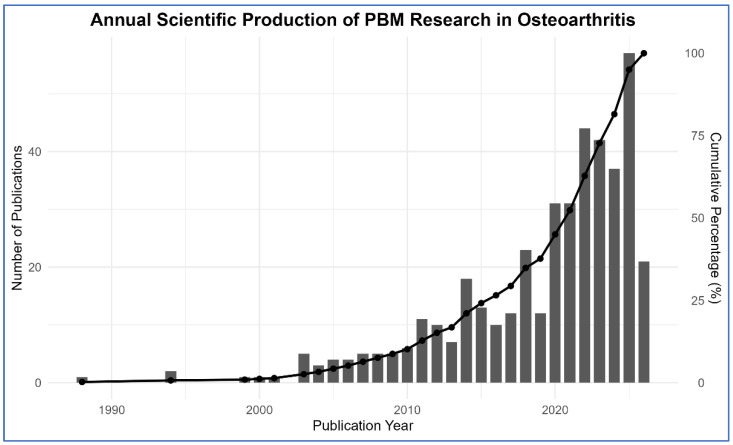
Annual scientific production of photobiomodulation research in osteoarthritis. Bars represent the number of annual publications related to photobiomodulation (PBM) and osteoarthritis (OA), while the black line indicates the cumulative publication percentage. The analysis included publications indexed in the Web of Science Core Collection from 1988 to 2026.

Figure 1 illustrates the annual publication trends of PBM and osteoarthritis research. Only 4 publications (0.9%) were published between 1988 and 1999, followed by 33 publications (7.8%) between 2000 and 2009 and 122 publications (28.9%) between 2010 and 2019. Publication output increased sharply during the most recent period, with 263 publications (62.3%) appearing between 2020 and 2026. Annual output increased from 1 publication in 1988 to 57 publications in 2025, corresponding to an overall annual growth rate of 8.34%. The 2026 count comprised 21 publications as of the search date and therefore represented a partial publication year.

Figure S3 and Table S4 present the leading journals publishing research on PBM and osteoarthritis. Lasers in Medical Science was the most productive journal, with 65 publications, followed by Photomedicine and Laser Surgery with 20 publications and Photobiomodulation, Photomedicine, and Laser Surgery with 11 publications. Journal of Clinical Medicine and Lasers in Surgery and Medicine each contributed 8 publications, while Clinical Rehabilitation and International Journal of Molecular Sciences each contributed 7 publications. Osteoarthritis and Cartilage, Clinical Rheumatology, BMC Musculoskeletal Disorders, Journal of Lasers in Medical Sciences, and Veterinary Clinics of North America: Small Animal Practice each contributed 6 publications (Table S4).

### 3.3. Keyword Co-Occurrence Network and Translational Research Structure

**Figure 2:**
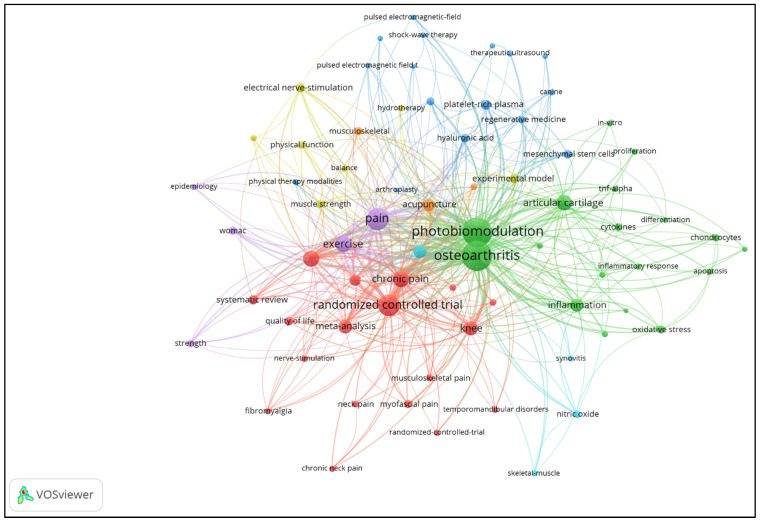
Keyword co-occurrence network of photobiomodulation and osteoarthritis research. The network was generated using VOSviewer based on author keywords and Keywords Plus. Node size represents keyword occurrence frequency, and link thickness indicates the strength of co-occurrence relationships. Different colors indicate distinct keyword clusters representing major research themes within the PBM-OA field.

The keyword co-occurrence network analysis classified a total of 71 keywords into seven clusters (Figure 2; Table S5). The largest clusters were Cluster 1 and Cluster 2, each containing 18 keywords, followed by Cluster 3 with 16 keywords, Cluster 4 with 7, Cluster 5 with 5, Cluster 6 with 4, and Cluster 7 with 3 keywords.

At the center of the network, “osteoarthritis” showed the highest occurrence frequency (348 occurrences) and total link strength (TLS = 1296), followed by “photobiomodulation” (338 occurrences; TLS = 1254). Other prominent network hubs included “pain” (141 occurrences; TLS = 606), “randomized controlled trial” (127 occurrences; TLS = 573), “articular cartilage” (67 occurrences; TLS = 262), “chronic pain” (64 occurrences; TLS = 280), “exercise” (60 occurrences; TLS = 289), “physical therapy” (60 occurrences; TLS = 276), and “knee” (47 occurrences; TLS = 213). These values indicate that the field was organized around two dominant disease-intervention concepts, with strong secondary connections to pain, clinical trial methodology, rehabilitation, and cartilage biology.

Cluster 1 comprised 18 keywords related to clinical trials, pain management, physical therapy, and clinical outcome assessment. Its most prominent terms were “randomized controlled trial” (127 occurrences; TLS = 573), “chronic pain” (64; TLS = 280), “physical therapy” (60; TLS = 276), “knee” (47; TLS = 213), and “meta-analysis” (38; TLS = 182).

Cluster 2 also contained 18 keywords and represented mechanistic pathways, inflammatory responses, and cartilage biology. The dominant terms were “osteoarthritis” (348; TLS = 1296), “photobiomodulation” (338; TLS = 1254), “articular cartilage” (67; TLS = 262), “inflammation” (38; TLS = 157), and “oxidative stress” (12; TLS = 53).

Cluster 3 comprised 16 keywords associated with regenerative medicine and adjunctive therapeutic modalities. Its leading terms included “platelet-rich plasma” (20 occurrences; TLS = 91), “mesenchymal stem cells” (13; TLS = 60), “hyaluronic acid” (13; TLS = 62), “extracorporeal shockwave therapy” (10; TLS = 53), and “regenerative medicine” (6; TLS = 39).

Cluster 4 comprised 7 keywords related to functional rehabilitation and physical performance. The most connected terms were “experimental model” (15 occurrences; TLS = 72), “electrical nerve-stimulation” (13; TLS = 69), “muscle strength” (8; TLS = 50), and “physical function” (8; TLS = 39).

Cluster 5 contained 5 keywords related to exercise, pain assessment, and functional outcomes, led by “pain” (141 occurrences; TLS = 606), “exercise” (60; TLS = 289), and “WOMAC” (12; TLS = 55).

Cluster 6 contained 4 keywords linking rehabilitation and biological signaling, including “rehabilitation” (39 occurrences; TLS = 160), “nitric oxide” (10; TLS = 39), “skeletal-muscle” (6; TLS = 20), and “synovitis” (5; TLS = 23).

Cluster 7 was the smallest cluster, comprising 3 keywords related to integrative and modality-specific interventions: “acupuncture” (30 occurrences; TLS = 148), “musculoskeletal” (9; TLS = 48), and “intensity laser therapy” (7; TLS = 32).

### 3.4. Temporal Evolution and Research Hotspots

**Figure 3:**
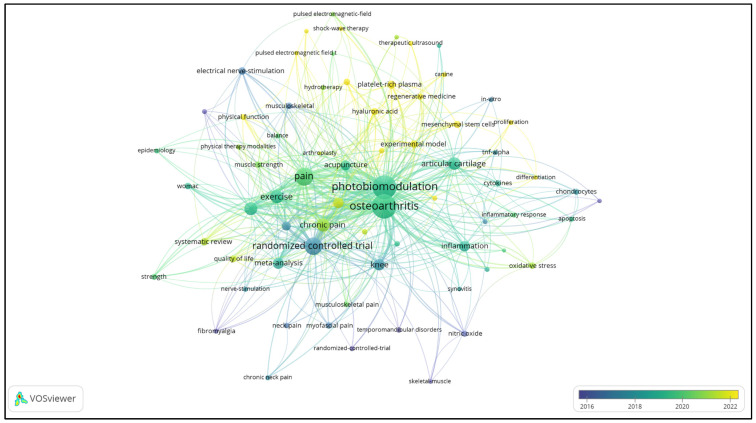
Overlay visualization of keyword evolution in PBM–osteoarthritis research. Overlay visualization was generated using VOSviewer based on the average publication year of keywords. Blue-colored nodes indicate relatively earlier research topics, whereas yellow-colored nodes represent more recent and emerging research themes.

The overlay visualization demonstrated a temporal shift from earlier rehabilitation- and symptom-oriented topics toward regenerative and technology-based research (Figure 3). Earlier keywords included “osteo-arthritis” (average publication year [APY] = 2003.6), “metabolism” (APY = 2011.4), “skeletal-muscle” (APY = 2014.5), “temporomandibular disorders” (APY = 2014.6), and “fibromyalgia” (APY = 2014.8). Intermediate-period topics included “electrical nerve-stimulation” (APY = 2017.0), “randomized controlled trial” (APY = 2017.5), “knee” (APY = 2017.7), “inflammation” (APY = 2018.9), “exercise” (APY = 2019.2), “photobiomodulation” (APY = 2019.3), and “osteoarthritis” (APY = 2019.5).

More recent topics included “platelet-rich plasma” (APY = 2022.7), “mesenchymal stem cells” (APY = 2022.8), “regenerative medicine” (APY = 2022.3), “extracorporeal shockwave therapy” (APY = 2023.0), “therapeutic ultrasound” (APY = 2023.2), “association” (APY = 2023.2), “intensity laser therapy” (APY = 2024.4), and “pulsed electromagnetic field therapy” (APY = 2024.6). These findings quantitatively support the emergence of regenerative medicine and advanced energy-delivery modalities as recent research directions.

The density visualization confirmed that “osteoarthritis” (348 occurrences), “photobiomodulation” (338), “pain” (141), and “randomized controlled trial” (127) formed the highest-density regions of the network (Figure S4). Secondary high-density areas were observed around “articular cartilage” (67), “chronic pain” (64), “exercise” (60), “physical therapy” (60), “knee” (47), “rehabilitation” (39), and “inflammation” (38).

### 3.5. Thematic Structure of PBM-OA Research

**Figure 4:**
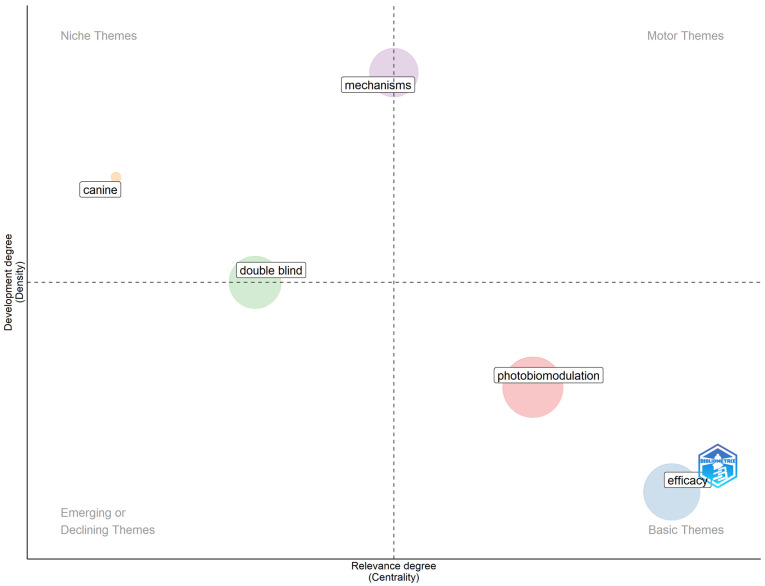
Thematic map of PBM–osteoarthritis research based on keyword centrality and density. The thematic map was generated using the bibliometrix package in R. Themes were classified into motor themes, basic themes, niche themes, and emerging/declining themes according to centrality (relevance degree) and density (development degree). Bubble size represents the relative frequency of each theme.

The thematic map analysis identified five principal thematic groups (Figure 4). “Mechanisms” was positioned within the motor themes quadrant, indicating comparatively high centrality and density. “Photobiomodulation” and “efficacy” were located within the basic themes quadrant, whereas “canine” was positioned within the niche themes quadrant. In addition, “double blind” was located near the central axis and exhibited moderate levels of centrality and density.

### 3.6. Intellectual Structure of the Field: Reference Co-Citation Network

**Figure 5:**
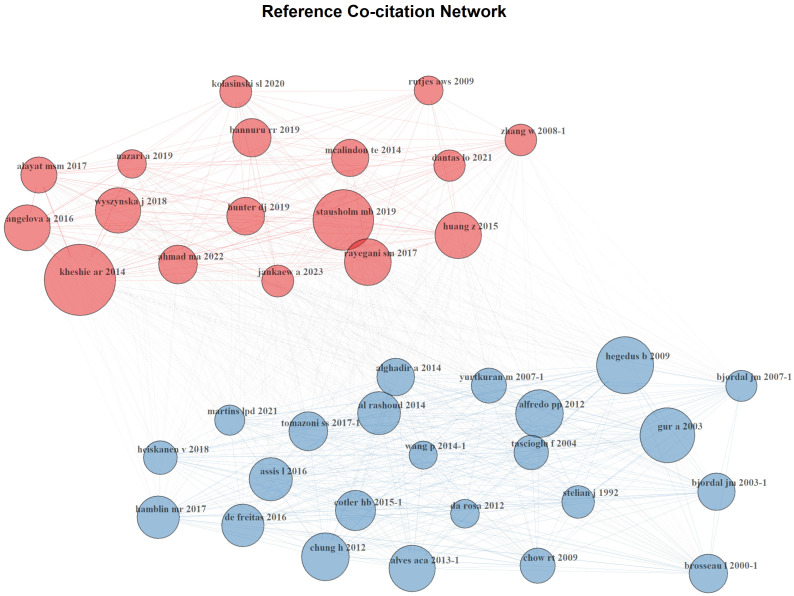
Reference co-citation network in PBM–osteoarthritis research. The co-citation network was constructed based on references co-cited within the included publications. Node size represents citation frequency, and link thickness indicates co-citation strength. Different colors indicate major intellectual clusters within the PBM-OA research field.

Reference co-citation network analysis revealed that the PBM-OA research field consisted of two major co-citation clusters (Figure 5). The first cluster was centered around studies by Kheshie et al. [8], Stausholm et al. [9], Rayegani et al. [10], and Huang et al. [11]. Among these, Kheshie et al. [8] had received 145 citations, Stausholm et al. [9] 114 citations, and Huang et al. [11] 101 citations within the bibliographic dataset.

The second cluster was centered around studies by Gur et al. [12], Hegedus et al. [13], Alfredo et al. [14], and Assis et al. [15], which had received 113, 128, 87, and 89 citations, respectively. Multiple linkages were observed between the two clusters. Studies related to pain management, inflammation, and rehabilitation appeared to connect the two clusters within the co-citation network.

## 4. Discussion

### 4.1. Overview of the Main Findings

The present bibliometric analysis demonstrates that photobiomodulation (PBM)-based osteoarthritis (OA) research has experienced sustained growth over the past two decades, with a marked expansion in both research volume and thematic diversity in recent years. Notably, publication output increased substantially after 2015, and the annual number of publications rose rapidly after 2020 (Figure 1). It is important to note that this quantitative growth reflects increasing scholarly attention and publication activity within the scientific community, rather than providing direct evidence of the clinical efficacy or therapeutic effectiveness of PBM in osteoarthritis.

Furthermore, the present study showed that the PBM-OA field has developed a translational research structure characterized by the concurrent growth of mechanistic biological research and clinical rehabilitation research. In both the keyword co-occurrence network and the reference co-citation network, a mechanistic biology axis represented by terms such as “inflammation,” “chondrocytes,” “oxidative stress,” and “cartilage” coexisted with a clinical rehabilitation axis centered on “pain,” “WOMAC,” “rehabilitation,” and “physical therapy,” together forming a major structural component of the field (Figure 2 and Figure 5).

In addition, thematic map analysis and overlay visualization suggest a shift in research focus within the PBM-OA literature, moving from early empirical evaluations of clinical efficacy toward increasing attention to mechanistic investigations and regenerative medicine-related topics. This pattern reflects the evolving intellectual landscape and research priorities of the field.

### 4.2. Translational Structure of PBM-OA Research

One of the most important findings of the present study is that PBM research in osteoarthritis has developed around two major research domains: mechanistic biology and clinical rehabilitation. In the keyword network, mechanistic terms such as “inflammation,” “oxidative stress,” “chondrocytes,” and “cartilage” showed high centrality alongside clinically oriented terms including “pain,” “WOMAC,” “rehabilitation,” and “physical therapy.” A similar dual structure was also observed in the reference co-citation network. This thematic organization suggests that the published literature has increasingly investigated PBM from both mechanistic and rehabilitation-oriented perspectives, reflecting the evolving intellectual landscape of the field.

This structure likely reflects the growing research interest in understanding the relationship between OA pathophysiology and the biological targets of PBM. Osteoarthritis is increasingly recognized as a complex disease involving chronic inflammation, oxidative stress, chondrocyte dysfunction, and extracellular matrix degradation rather than a condition caused solely by cartilage wear [16,17,18], and the bibliometric findings indicate that research attention has increasingly focused on these pathophysiological processes. At the same time, the prominence of rehabilitation-related terms suggests that these mechanistic themes are increasingly being investigated alongside clinically relevant outcomes such as pain, function, and rehabilitation [9,19].

The structures observed in Figure 2 and Figure 5 reflect how a bench-to-bedside paradigm is represented within the academic discourse, linking mechanistic investigations of OA pathophysiology with rehabilitation-oriented research. Collectively, these findings suggest that the PBM-OA literature has progressively expanded from symptom-oriented research to encompass a broader conceptual framework integrating mechanistic biology and rehabilitation science.

### 4.3. Evolution from Symptom Management to Regenerative Medicine

The overlay visualization illustrates how research interests within the PBM-OA field have evolved over time. Early studies were primarily characterized by keywords such as “pain,” “WOMAC,” “physical function,” and “rehabilitation,” suggesting that published studies primarily investigated PBM as an adjunctive rehabilitation intervention for pain relief and functional recovery [12,14]. This pattern is consistent with the clinical emphasis of OA management during that period, which focused primarily on symptom control and maintenance of physical function rather than structural restoration.

Subsequently, the increasing prominence of keywords related to “randomized controlled trial” and “efficacy” reflected growing efforts to evaluate the clinical effectiveness of PBM. Systematic reviews and meta-analyses began to synthesize evidence regarding pain reduction and functional improvement while also examining differences according to wavelength, dose, and treatment schedule [9,19]. This trend reflects a progression within the published literature from empirical application toward evidence-based investigation.

More recently, keywords such as “regenerative medicine,” “mesenchymal stem cells,” “platelet-rich plasma,” “hydrogel,” and “experimental model” have emerged, indicating increasing scholarly interest in tissue repair and regenerative medicine-related approaches. While conventional OA treatments remain largely focused on symptom management, recent studies have increasingly explored tissue regeneration and structural restoration [20], and the bibliometric findings indicate that PBM has increasingly been investigated within this broader research context.

Within the present dataset, this research trend is reflected by the emergence of studies investigating PBM in combination with biological regenerative approaches. Specifically, publications evaluating PBM together with platelet-rich plasma [21,22], or mesenchymal stem cells [23,24], have formed prominent thematic clusters in recent years, reflecting increasing research interest in exploring their potential synergistic effects on cartilage preservation and tissue protection.

Collectively, these bibliometric patterns indicate that the conceptual orientation of the PBM-OA literature is gradually expanding beyond symptom-focused rehabilitation research toward increasing scholarly attention to mechanistic investigations and regenerative medicine-related approaches, reflecting the evolving research priorities of the field.

### 4.4. Emerging Mechanistic Focus and Field Maturity

The positioning of “mechanisms” as a motor theme and “efficacy” as a basic theme in the thematic map provides insight into the academic maturity of the PBM-OA field. This pattern suggests that the focus of research is no longer limited to whether PBM is effective, but is increasingly directed toward understanding the biological mechanisms through which PBM exerts its effects.

Within this thematic structure, the PBM literature shows increasing attention to cellular and molecular pathways. Foundational studies have discussed mitochondrial photoacceptor mechanisms centered on cytochrome c oxidase, with red and near-infrared light investigated in relation to ATP production, reactive oxygen species (ROS) modulation, and nitric oxide signaling. These processes have further been linked to NF-κB inhibition, Nrf2 activation, inflammatory regulation, and cell-survival pathways [25,26,27]. The prominence of these mechanistic topics reflects the increasing specialization of the field’s intellectual structure.

This mechanistic orientation is also consistent with the pathological processes investigated in experimental OA models. Published studies have examined PBM in relation to matrix metalloproteinase activity, type II collagen preservation, inflammatory cytokine regulation, and cartilage integrity [20,28,29]. The convergence of these topics within the literature indicates that mechanistic investigations have become an increasingly prominent component of PBM-OA research.

Therefore, the emergence of “mechanisms” as a motor theme reflects more than a simple change in keyword frequency. It indicates that the conceptual structure of the PBM-OA literature is expanding from empirical efficacy evaluation toward a broader and more integrated emphasis on mechanistic investigation.

### 4.5. Clinical Implications for Osteoarthritis Rehabilitation

The high centrality of “physical therapy,” “exercise,” “WOMAC,” and “rehabilitation” within the keyword network shows a clear trend. It indicates that the published literature examines PBM within broader rehabilitation strategies rather than as a stand-alone intervention. This observation aligns with current clinical guidelines. While exercise, weight management, education, and self-management remain core components of conservative OA management, PBM is positioned as a complementary non-pharmacological intervention [30,31].

In particular, the persistent prominence of “exercise,” “rehabilitation,” and “WOMAC” in Figure 2 suggests that the clinical application of PBM has primarily been investigated in conjunction with rehabilitation programs. Patients with OA frequently experience pain-related reductions in exercise capacity and adherence, contributing to progressive declines in physical function. Accordingly, the close thematic association between PBM and exercise-related concepts within the keyword network reflects sustained research interest in evaluating whether PBM may facilitate participation in exercise-based rehabilitation and improve rehabilitation-related outcomes.

This interpretation is supported by the tendency of published clinical studies to evaluate PBM in combination with strengthening exercise, therapeutic exercise, or physical therapy rather than as a stand-alone intervention [32,33]. Recent meta-analyses have similarly examined whether combining PBM with exercise provides additional benefits for pain reduction and functional improvement, although the reported findings remain inconsistent [34,35,36].

Collectively, these findings suggest that the current PBM-OA literature predominantly positions PBM as an adjunctive rehabilitation tool. This underscores the focus of current research on supporting active, exercise-centered conservative management.

### 4.6. Study Limitations

The findings of the present study should be interpreted in light of several limitations. First, the bibliometric dataset was derived exclusively from the Web of Science Core Collection (WoSCC). Although WoSCC provides highly standardized bibliographic metadata that facilitate co-citation analysis, keyword co-occurrence analysis, and thematic mapping, publications indexed exclusively in other databases, such as Scopus, PubMed, or EMBASE, were not included.

Furthermore, because this study integrated both Author Keywords and WoSCC-specific Keywords Plus to expand keyword coverage and enhance network robustness, utilizing a single standardized database was methodologically preferable to combining multiple databases with disparate indexing systems and metadata structures. This approach was deemed appropriate given that the primary objective of the present study was to characterize the intellectual structure and research landscape of the field, rather than to achieve comprehensive literature retrieval.

Nevertheless, exclusive reliance on a single database may have resulted in incomplete coverage of the broader literature. Future bibliometric studies integrating multiple databases using harmonized metadata frameworks may provide a more comprehensive representation of this research field.

### 4.7. Conclusions

This bibliometric analysis provides a comprehensive overview of the evolution and intellectual structure of PBM research in osteoarthritis over the past several decades. The findings reveal a progressive diversification of research themes, with the literature increasingly encompassing mechanistic biology, regenerative medicine-related approaches, and rehabilitation-oriented applications.

To strengthen the evidence base, future research should prioritize protocol standardization, rigorous dose–response validation, and long-term clinical studies incorporating objective biomarkers. Concurrently, emerging topics such as regenerative combination therapies and precision PBM technologies represent promising directions for future investigation.

Overall, this study provides a bibliometric framework for understanding the current knowledge structure of the PBM-OA field, identifying emerging research priorities, and guiding future investigations in this rapidly evolving area.

## Data Availability

The data supporting the findings of this study are available from the corresponding author upon reasonable request.

## Supplementary Materials

The following supporting information can be downloaded at: https://www.mdpi.com/article/10.3390/bioengineering13070811/s1.
