# Supplementary material for: Mapping the Translational Research Structure of Photobiomodulation in Osteoarthritis: A Bibliometric Analysis
Source: Bioengineering (Basel). 2026 Jul 15;13(7):811. doi: 10.3390/bioengineering13070811 (PMC13405207; doi:10.3390/bioengineering13070811)
Supplement: Supplementary file 1 [file bioengineering-13-00811-s001.zip › bioengineering-4400347-supplementary.pdf]

Table S1. Keyword normalization and filtering scheme

Original keyword	Category	Action	Reason
low-level laser therapy	synonym	merged into “photobiomodulation”	standardized PBM terminology
low level laser therapy	synonym	merged into “photobiomodulation”	standardized PBM terminology
LLLT	abbreviation	merged into “photobiomodulation”	abbreviation normalization
PBMT	abbreviation	merged into “photobiomodulation”	abbreviation normalization
PBM	abbreviation	merged into “photobiomodulation”	abbreviation normalization
laser therapy	synonym	merged into “photobiomodulation”	standardized intervention terminology
phototherapy	synonym	merged into “photobiomodulation”	standardized intervention terminology
light-emitting diode	device-related synonym	merged into “photobiomodulation”	terminology standardization
knee osteoarthritis	disease synonym	merged into “osteoarthritis”	disease normalization
osteoarthrosis	disease synonym	merged into “osteoarthritis”	disease normalization
degenerative joint disease	disease synonym	merged into “osteoarthritis”	disease normalization
cartilage	anatomical synonym	merged into “articular cartilage”	improved semantic consistency
double blind	methodological synonym	merged into “randomized controlled trial”	methodological harmonization
placebo-controlled trial	methodological synonym	merged into “randomized controlled trial”	methodological harmonization
physiotherapy	synonym	merged into “physical therapy”	terminology consistency
exercise therapy	synonym	merged into “physical therapy”	terminology consistency
platelet rich plasma	synonym	merged into “platelet-rich plasma”	standardized regenerative medicine terminology
shock wave therapy	synonym	merged into “shock-wave therapy”	terminology consistency
rheumatoid arthritis	non-target disease	removed	outside OA scope
therapy	generic term	removed	generic intervention term that does not distinguish specific

			research topics
treatment	generic term	removed	generic intervention term lacking thematic specificity
management	generic term	removed	broad intervention descriptor lacking conceptual specificity
disease	generic term	removed	overly broad descriptor not specific to PBM-OA research
efficacy	non-specific outcome term	removed	non-specific outcome descriptor
outcomes	generic term	removed	generic outcome descriptor
model	generic term	removed	generic methodological descriptor
rats	animal descriptor	removed	animal descriptor rather than a research theme
women	demographic descriptor	removed	demographic descriptor rather than a thematic concept
function	generic descriptor	removed	broad functional descriptor lacking thematic specificity

Table S2. Keyword thesaurus for normalization and exclusion in the VOSviewer co-occurrence network analysis

Original keyword	Standardized term	Action
low-level laser therapy	photobiomodulation	merged
low level laser therapy	photobiomodulation	merged
low-level light therapy	photobiomodulation	merged
laser therapy	photobiomodulation	merged
phototherapy	photobiomodulation	merged
LLLT	photobiomodulation	merged
PBMT	photobiomodulation	merged
PBM	photobiomodulation	merged
knee osteoarthritis	osteoarthritis	merged
osteoarthrosis	osteoarthritis	merged
degenerative joint disease	osteoarthritis	merged
cartilage	articular cartilage	merged
pain relief	chronic pain	merged
analgesia	chronic pain	merged
double blind	randomized controlled trial	merged

placebo-controlled trial	randomized controlled trial	merged
physiotherapy	physical therapy	merged
exercise therapy	physical therapy	merged
platelet rich plasma	platelet-rich plasma	merged
shock wave therapy	shock-wave therapy	merged
therapy	N.A.	removed
treatment	N.A.	removed
management	N.A.	removed
disease	N.A.	removed
efficacy	N.A.	removed
outcomes	N.A.	removed
model	N.A.	removed
women	N.A.	removed
rats	N.A.	removed
function	N.A.	removed

Table S3. General bibliometric characteristics of PBM-OA publications

Characteristic	Value
Database	Web of Science Core Collection (WoSCC)
Search date	May 24, 2026
Initial retrieved records	503
Records after document type filtering	433
Final included publications	422
Duplicate records removed	0
Publication years	1988–2026
Total citations	9,497
Average citations per document	22.5
Total journals	198
Average authors per paper	6.24
Document types	Article: 305, Review: 117

Table S4. Top journals publishing PBM-OA research

Rank	Journal	Publications
1	Lasers in Medical Science	65
2	Photomedicine and Laser Surgery	20
3	Photobiomodulation, Photomedicine and Laser Surgery	11
4	Lasers in Surgery and Medicine	8
5	Journal of Clinical Medicine	8
6	Clinical Rehabilitation	7
7	International Journal of Molecular Sciences	7
8	Veterinary Clinics of North America-Small Animal Practice	6
9	Clinical Rheumatology	6
10	BMC Musculoskeletal Disorders	6
11	Journal of Lasers in Medical Sciences	6
12	Osteoarthritis and Cartilage	6
13	Journal of Photochemistry and Photobiology B-Biology	5

14	Biomedicines	4
15	Photochemistry and Photobiology	4
16	Journal of Back and Musculoskeletal Rehabilitation	4
17	Medicine	3
18	American Journal of Physical Medicine & Rehabilitation	3
19	Frontiers in Cell and Developmental Biology	3
20	BMJ Open	3

Table S5. Major keyword clusters identified in the co-occurrence network analysis

Cluster	Keywords in cluster	Research theme
Cluster 1 (18 items)	chronic neck pain; chronic pain; fibromyalgia; knee; meta-analysis; musculoskeletal disorders; musculoskeletal pain; myofascial pain; neck pain; nerve-stimulation; physical therapy; quality of life; randomized controlled trial; randomized-controlled-trial; systematic review; temporomandibular disorders; tendinopathy; ultrasound	Clinical trials, pain management, and physical therapy
Cluster 2 (18 items)	apoptosis; articular cartilage; association; chondrocytes; cytokines; differentiation; gene expression; in-vitro; inflammation; inflammatory response; metabolism; osteoarthritis; osteochondral defect; oxidative stress; photobiomodulation; proliferation; temporomandibular joint; TNF-alpha	Mechanistic, inflammatory, and cartilage biology
Cluster 3 (16 items)	arthroplasty; canine; continuous passive motion; extracorporeal shockwave therapy; hyaluronic acid; intensity pulsed ultrasound; mesenchymal stem cells; neuropathic pain; physical therapy modalities; platelet-rich plasma; postoperative pain; pulsed electromagnetic field therapy; pulsed electromagnetic-field; regenerative medicine; shock-wave therapy; therapeutic ultrasound	Regenerative medicine and adjunctive therapeutic modalities
Cluster 4 (7 items)	balance; electrical nerve-stimulation; experimental model; hydrotherapy; muscle strength; osteo-arthritis; physical function	Functional rehabilitation and physical performance
Cluster 5 (5 items)	epidemiology; exercise; pain; strength; WOMAC	Exercise, pain assessment, and functional outcomes
Cluster 6 (4 items)	nitric oxide; rehabilitation; skeletal-muscle; synovitis	Rehabilitation-linked biological signaling
Cluster 7 (3 items)	acupuncture; intensity laser therapy; musculoskeletal	Integrative and modality-specific interventions

Figure S1. Flow diagram of literature selection for bibliometric analysis.

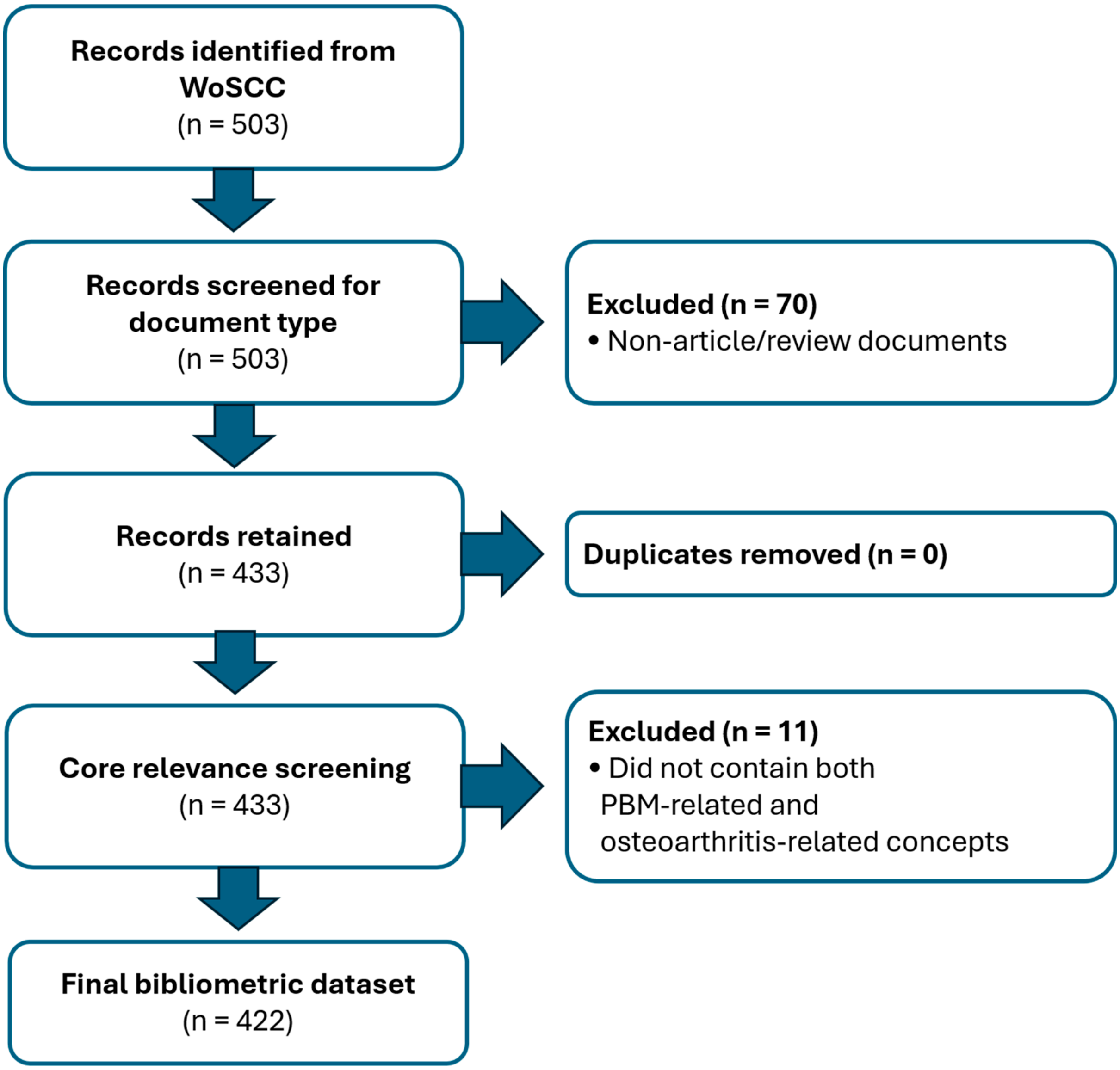

Figure S2. Preclinical–clinical orientation of PBM-osteoarthritis studies.

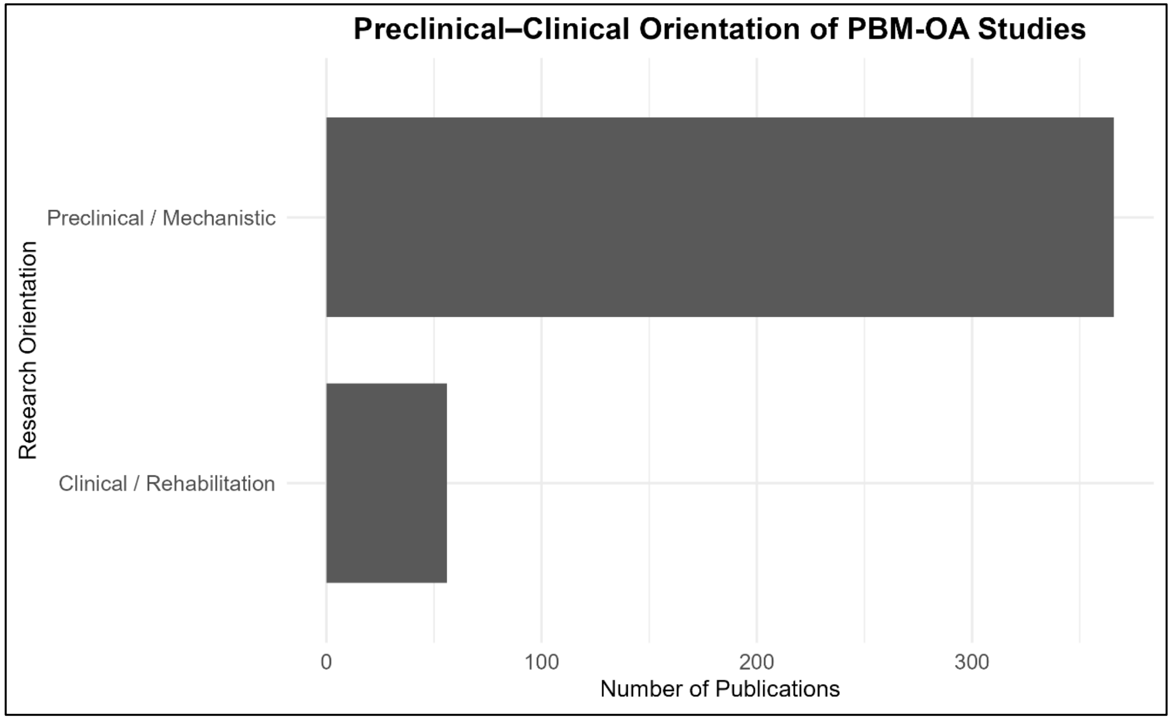

The figure illustrates the distribution of publications according to research orientation. Studies were classified into preclinical/mechanistic research and clinical/rehabilitation research based on title, abstract, and keyword content.

Figure S3. Top journals publishing PBM-osteoarthritis research.

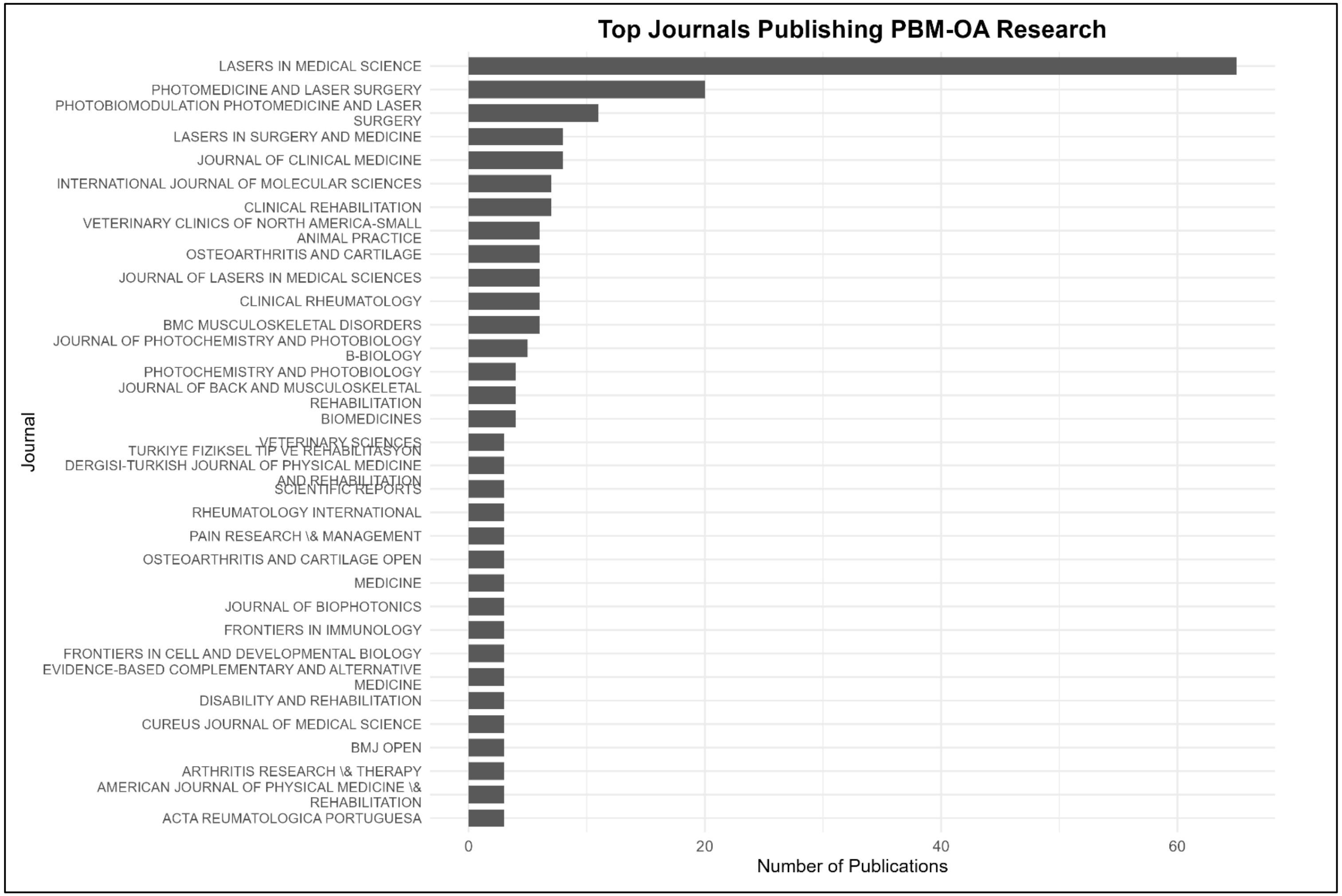

The figure presents the leading journals contributing to PBM-osteoarthritis research based on publication frequency. Journal counts were calculated using bibliographic data extracted from the Web of Science Core Collection.

Figure S4. Density visualization of keyword co-occurrence in PBM-osteoarthritis research.

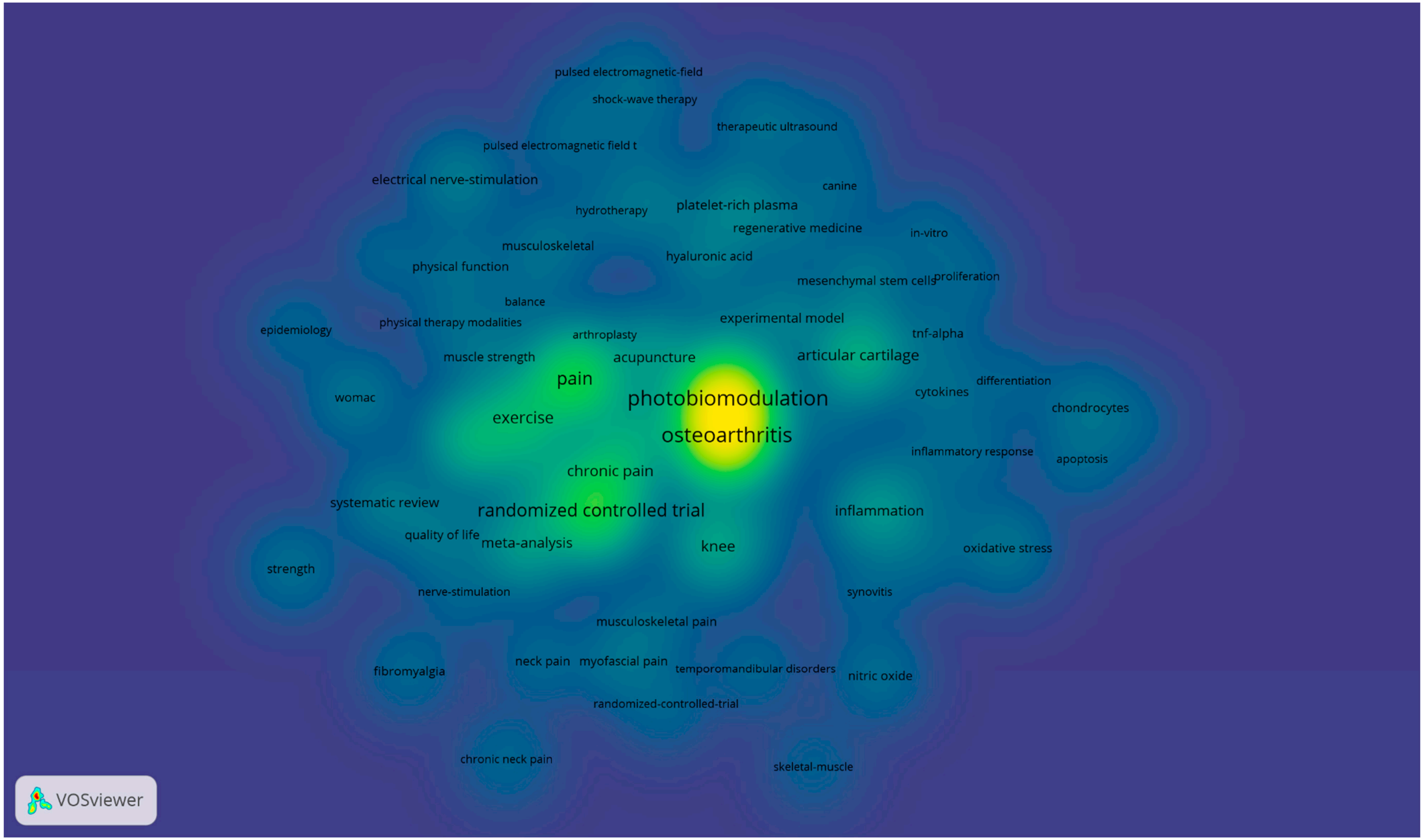

Density visualization was generated using VOSviewer. Regions with warmer colors (yellow) indicate higher keyword occurrence frequency and stronger connectivity, representing major research hotspots within the field.
